# Wide-Line NMR Melting Diagrams, Their Thermodynamic Interpretation, and Secondary Structure Predictions for A30P and E46K α-Synuclein

**DOI:** 10.1021/acsomega.2c00477

**Published:** 2022-05-23

**Authors:** Mónika Bokor, Eszter Házy, Ágnes Tantos

**Affiliations:** † Institute for Solid State Physics and Optics, Wigner Research Centre for Physics, 1121 Budapest, Hungary; ‡ Institute of Enzymology, Research Centre for Natural Sciences, 1117 Budapest, Hungary

## Abstract

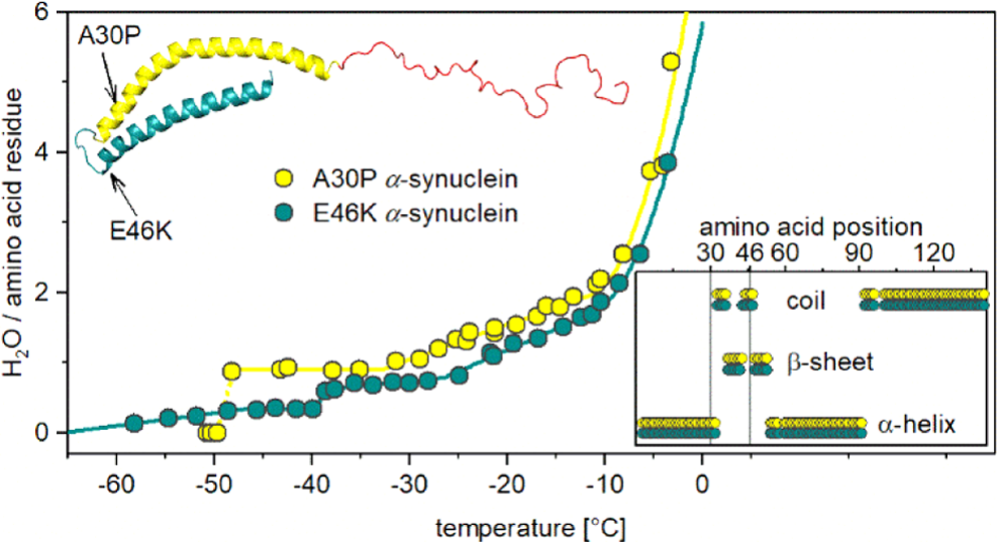

Parkinson’s disease is thought to be caused by aggregation of the intrinsically disordered protein, α-synuclein. Two amyloidogenic variants, A30P, and E46K familial mutants were investigated by wide-line ^1^H NMR spectrometry as a completion of our earlier work on wild-type and A53T α-synuclein (BokorM. et al. WT and A53T α-synuclein systems: melting diagram and its new interpretation. Int. J. Mol. Sci.2020, 21, 3997.10.3390/ijms21113997PMC731260132503167). A monolayer of mobile water molecules hydrates A30P α-synuclein at the lowest potential barriers (temperatures), while E46K α-synuclein has here third as much mobile hydration, insufficient for functionality. According to wide-line ^1^H NMR results and secondary structure predictions, E46K α-synuclein is more compact than the A30P variant and they are more compact than the wild type (WT) and A53T variants. Linear hydration *vs* potential barrier sections of A30P α-synuclein shows one and E46K shows two slopes. The different slopes of the latter between potential barriers *E*_a,1_ and *E*_a,2_ reflect a change in water–protein interactions. The 31–32% homogeneous potential barrier distribution of the protein–water bonds refers to a non-negligible amount of secondary structures in all four α-synuclein variants. The secondary structures detected by wide-line ^1^H NMR are solvent-exposed α-helices, which are predicted by secondary structure models. β-sheets are only minor components of the protein structures as three- and eight-state predicted secondary structures are dominated by α-helices and coils.

## 1 Introduction

The intrinsically disordered proteins (IDPs) are unfolded under physiological conditions. While this feature is important for many physiological functions,^1^ disordered sequences are often prone to aggregation and fibril formation with detrimental consequences.^2^ Several neurodegenerative diseases are caused by the abnormal oligomerization and polymerization of proteins with disordered regions, including Alzheimer’s disease and Parkinson’s disease (PD). The exact molecular event that induces pathological aggregation of these proteins is still waiting to be elucidated,^3^ but there are certain verified mutations that render disordered proteins more prone to aggregation than their wild-type counterparts. α-Synuclein (α-S) is an IDP under normal physiological conditions (it is the general consensus),^4^ adopting random coil conformation.^5,6^ Notwithstanding, there exist variations of wild-type (WT) α-S structural propensity (globular or extended). The suppositions about secondary and tertiary structures of α-S are highly controversial, including conflicting views in the literature.^7^ The rapid interconversion between conformers impacting α-S and the different experimental methodologies used can be the causes of the controversies. α-S is coded by the SNCA gene, with several point mutations in the gene known to cause familial forms of PD, including A30P, A53T, and E46K mutations.^8^ A30P mutation retards^9^ the formation of both oligomers and fibrils and only this mutation affects the overall α-S structure.^7^ The E46K mutant increases membrane affinity^10^ and accelerates α-S aggregation and fibril formation.^11,12^ The A53T mutant considerably accelerates α-S aggregation, and its fibril formation is faster than that of the wild-type (WT) α-S.^13−15^

Different research groups came to different conclusions about α-S monomers having a globular-like structure or being extended random coils.^7^ Earlier, the WT and A53T α-S variants were investigated in monomer, oligomer, and amyloid forms.^16^ The monomers proved to be IDPs and more compact than random coils; about 32(3)% of their solvent-accessible surface is determined by the secondary structure. They are already functional at the lowest potential barriers with mobile hydration water: a monolayer of mobile hydration water is surrounding them. They realize all possible hydrogen bonds with the solvent water.

Wide-line ^1^H NMR is an accepted method that can provide information on the location and structural environment of hydrogen atoms in proteins as it enables the direct observation of translational and rotational movements of molecules in the condensed phase.

NMR characteristics of aqueous solutions rapidly frozen and then slowly thawed through equilibrium thermal states provide direct information on the immobile and partially or fully mobile parts of the molecules, yielding invaluable insight into the overall structure of the proteins.

Here, our previous work on WT and A53T α-S was completed with new results on two other familial mutants, A30P and E46K α-S. We measured the melting diagrams (MDs, relative ratios of mobile water as a function of normalized functional temperature; see the Supporting Information of ref (17)) by wide-line NMR, *i.e*., the amount of mobile hydration water as a function of temperature, to get information on the steps and gradience in the development of full hydration of α-S. Secondary structure (SS) predictions on the same proteins were calculated and compared to the experimental results by wide-line NMR. These measurements were supplemented with secondary structure predictions.

## 2 Materials and Methods

Expression and purification of recombinant human A30P and E46K mutant variants of α-S in a pRK-172-based expression system were performed as described.^18^ Briefly, expression of the proteins was performed in *Escherichia coli* Bl21(DE3) in a pT7-7-based expression system, after IPTG induction. Bacterial cell pellets were harvested by centrifugation and resuspended in 10 mM Tris–HCl, pH 8.0, 1 mM EDTA, and 1 mM cOmplete protease inhibitor cocktail. After cell lysis, streptomycin sulfate-precipitated DNA was removed by centrifugation and an ammonium sulfate precipitation step was performed to selectively precipitate the α-S protein. After centrifugation at 13,500*g* for 30 min at 4 °C, the pellet was dissolved in 10 mM Tris–HCl, pH 7.4, and 1 mM cOmplete, and filtered using a 0.2 μm mesh. The resulting solution was loaded onto a Resource Q anion exchange column on an Äkta Explorer chromatography system (GE Healthcare). The purity and integrity of the purified proteins were confirmed by SDS-PAGE (for representative gel pictures, see Supporting Figure 1). Peak fractions were collected and dialyzed against double-distilled water before lyophilization.

In sample preparation, the mass of lyophilized protein (without any further refinement) was measured, and an appropriate amount of double-distilled water was added to obtain the nominal concentration of 50 mg/mL. Oligomers formed during the process were removed by filtering the solution through a 100 kDa membrane. All measurements were carried out on three identical samples prepared independently.

The wide-line NMR approach we applied is detailed in ref (19). The beginning of the movement (rotation) of water molecules bound to the surface of the protein, the process that is considered melting, is followed by observing motional narrowing in wide-line ^1^H NMR spectroscopy. The motional narrowing is a useful criterion for mobility since dynamics of hydration water happens on a picosecond time scale and NMR is slower by an order of magnitude. Fundamental temperature is *T*_f_ = *R*·*T* and the normalized fundamental temperature scale is *T*_fn_ = *R*·*T*/(*R*·273.15) = *T*/273.15. The events of the beginning of molecular motion can be characterized on an energy scale by the application of *T*_fn_.

The number of water molecules in the first hydrate shell of the protein is *n*_ho_ and the total number of water molecules in the entire heterogeneous hydration region is *n*_he_. Applying these definitions, the total number of hydrating water molecules at 0 °C or *T*_fn_ = 1 is (*n*_ho_ + *n*_he_).

The number of mobile water molecules per amino acid residue is indicated with *naa*. It can be calculated from the measured fraction of mobile protons in water *n* as *naa* = (*n*/2)·(*M*_r_(protein)/*M*_r_(H_2_O))/*a*, where *a* is the number of amino acid residues in the protein and *M*_r_(*i*) is the relative formula mass of compound *i*.

The amount of mobile hydration can be given also by the common measure of hydration (not the same as the term mobile hydration) as the mass of the solvent water divided by the mass of the solute protein, *h* = *m*^water^/*m*^protein^. In our measurements, it is given by the measured (by wide-line ^1^H NMR) fraction of mobile water *n* multiplied by the mass of water and divided by the mass of protein, *h*^NMR^ = *n*·*m*^water^/*m*^protein^. Hydration and *naa* can be interrelated as *a.

The melting curve can be formally described as a series expansion^20,21^
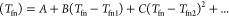
1where the summation is carried out up to the quadratic term. The cubic term, which was applied in ref (16), was unnecessary. *T*_fn0_ is the lowest temperature where mobile water molecules are detected and *E*_a,0_ (*T*_fn0_) is the lowest potential barrier with mobile water molecules at the solvent-accessible surface. The parameter *T*_fn1_ gives the temperature where the thermal trend of the MD switches between constant and linearly increasing. Likewise, the trend changes from linear to quadratic at *T*_fn2_.

Protein preparation and wide-line NMR measurements were described in former publications (ref (16) and the Supporting Information in ref (17)). The applied three-state SS prediction methods are Brewery, Jpred4, Porter 5.0, PSIPRED, PSRSM, RaptorX, SCRATCH, and SPIDER3. Further, eight-state predictions were made with the following methods only: Brewery, Porter 5.0, RaptorX, SCRATCH, and SPIDER3. Secondary structures made of a minimum of four amino acids consecutively are considered only.

Brewery^22^ (http://distilldeep.ucd.ie/brewery/) is the state-of-the-art predictor of protein structural annotations (secondary structure in three and eight classes). Brewery is based on ensembles of cascaded BRNNs (bidirectional recurrent neural networks) and Convolutional Neural Networks.

JPred is a protein secondary structure prediction server and has been in operation since approximately 1998. JPred incorporates the Jnet algorithm to make predictions that are more accurate. JPred4^23^ (http://www.compbio.dundee.ac.uk/jpred4/index.html) is its the current version.

Porter 5.0 and Porter8 5.0^24,25^ (http://distilldeep.ucd.ie/porter/) are servers for protein secondary structure prediction in three and eight classes based on ensembles of cascaded BRNNs and Convolutional Neural Networks.

PSIPRED^26^ (http://bioinf.cs.ucl.ac.uk/psipred/) is a simple and accurate secondary structure prediction method, incorporating two feed-forward neural networks, which perform an analysis on output obtained from PSI-BLAST (Position-Specific Iterated—BLAST).

PSRSM^27^ (http://qilubio.qlu.edu.cn:82/protein_PSRSM/default.aspx) uses methods based on data partitioning and the semirandom subspace method.

RaptorX Property^28^ (http://raptorx.uchicago.edu/) is a web server that predicts the structural properties of a protein sequence without using any templates. This server employs a powerful in-house deep-learning model, DeepCNF (Deep Convolutional Neural Fields), to predict the SS.

SCRATCH^29^ (http://scratch.proteomics.ics.uci.edu/) uses ensembles of bidirectional recurrent neural network architectures, PSI-BLAST-derived profiles, and a large nonredundant training set to derive two new predictors: (a) the second version of the SSpro program for secondary structure classification into three categories and (b) the first version of the SSpro8 program for secondary structure classification into the eight classes produced by the DSSP program.

SPIDER3^30^ (https://sparks-lab.org/server/spider3/) captures nonlocal interactions by long short-term memory bidirectional recurrent neural networks for improving the prediction of the protein secondary structure.

IUPred2A^31−35^ was used, which is a combined web interface that allows one to identify disordered protein regions using IUPred2 and disordered binding regions using ANCHOR2. Both IUPred2 and ANCHOR2 indicate a disordered region and a disordered binding site, respectively, with scores above 0.5.

## 3 Results

### 3.1 Direct Observations of Structural States through the Melting Diagrams

In an attempt to decipher the molecular background of the differences in the behavior of the two different α-S mutant variants, we conducted wide-line NMR measurements under identical conditions. In wide-line NMR, IDPs and globular proteins are easily distinguishable based on their MDs. While globular proteins show a plateau of a constant level of mobile hydration water throughout a relatively wide temperature range, disordered proteins are characterized by a constant growth in mobile water with increasing temperature.^19,20,36,37^ This measurement enables us to directly detect the mobility of the hydration layer surrounding the proteins, providing important insight into their structural flexibility. The most informative values are: *T*_fn0_, *t*_0_, or *E*_a,0_. *T*_fn0_ is the lowest functional temperature, where mobile hydration water appears; *t*_0_ is the same in degree Celsius units, and *E*_a,0_ is the corresponding potential barrier value calculated by multiplying with the special heat of ice at 0 °C.

**Figure 1:**
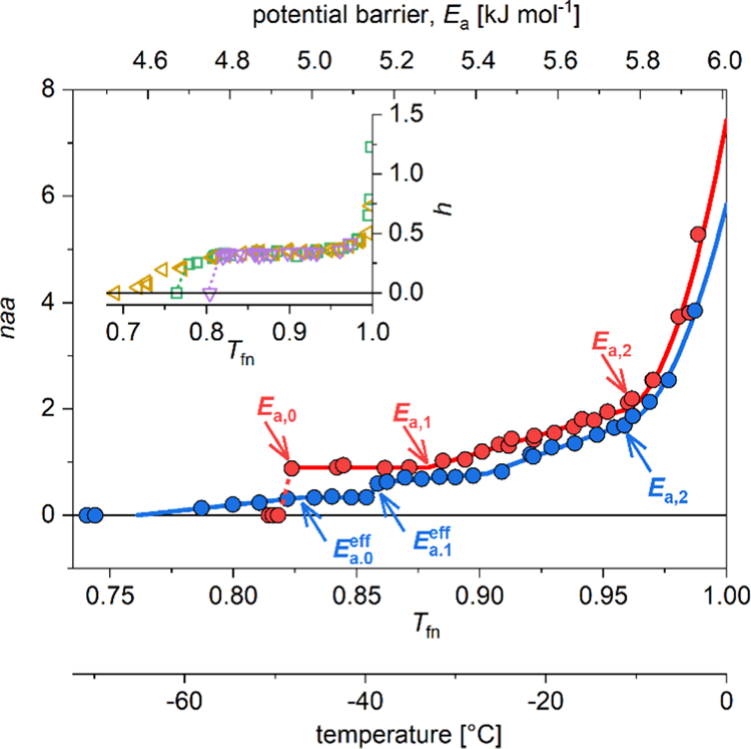
Melting diagram of A30P (red) and E46K (blue) α-synuclein. Trend changing points (*E*_a,i_) are indicated. Lines are fitted eq 1, and the parameters are given in Table 1. Inserted graph: hydration of the globular proteins, BSA (green squares), β-casein (violet down triangles), and lysozyme (gold left triangles) are plotted on the insert.

The MDs of A30P and E46K α-S (Figure 1) are characteristic of IDPs. They show the first constant amount of mobile hydration water at relatively high temperature/functional normalized temperature or potential barrier^19^ (*T*_fn0_, *t*_0_, or *E*_a,0_, Table 1) compared to globular proteins (see the Supporting Information of ref (17)), and they have an intensely elevating section from *E*_a,1_ on, in contrast to the constant number of mobile water molecules per amino acid residue, *naa* (see Materials and Methods) values of globular proteins in the same temperature range.

**Table 1:** Parameter Values for the Polynomial Relation (eq 1 Describes the Mobile Water Fraction, *n*)^a^

α-S variant	A30P	E46K
*A* = *n*(*T*_fn0_) = *n*(*T*_fn1_)^b^	0.0156(2)	0.0058(1)
*naa*(*E*_a,0_) = *naa*(*E*_a,1_)	0.90(1)	0.335(6)
*h*(*E*_a,0_) = *h*(*E*_a,1_)	0.313(4)	0.117(2)
*B*	0.24(1)	0.07(1), 0.29(2)^c^
*C*	5(1)·10^1^	36(5)
*T*_fn0_^b^	0.824(3)	0.828(3)
*E*_a,0_/kJ mol^–1^	4.95(2)	4.97(2)
*t*_0_/°C	–48.2(8)	–47.1(8)
*T*_fn1_, *T*_fn1_^eff^	0.879(2)	0.875(4), 0.856(2)
*E*_a,1_, *E*_a,1_^eff^/kJ mol^–1^	5.28(1)	5.26(2), 5.15(1)
*t*_1_, *t*_1_^eff^/°C	–33.0(7)	–34(1), −39.4(6)
*T*_fn2_	0.959(4)	0.959(2)
*E*_a,2_/kJ mol^–1^	5.76(2)	5.76(1)
*t*_2_/°C	–11(1)	–11.2(5)
*n*(*T*_fn2_)	0.0345(2)	0.030(2)
*naa*(*E*_a,2_)	1.98(1)	1.7(1)
*h*(*E*_a,2_)	0.689(4)	0.60(3)
*n*(*T*_fn_ = 1)	0.13(2)	0.102(2)
*naa*(*E*_a_ = 6.01 kJ mol^–1^)	7(1)	5.9(9)
*h*(*E*_a_ = 6.01 kJ mol^–1^)	2.6(4)	2.0(3)

a Error in the last digit is given in parentheses. *n*(*i*) values are given for a 50 mg/mL protein concentration.

b *T*_fn0_^eff^ in the case of E46K.

c Below and above *T*_fn_ = 0.903, respectively.

If we want to understand the differences in the behavior of the two mutant proteins, the best way is to create a differential melting diagram (DMD) by plotting the differential values calculated from the MDs against functional temperature (Δ*n*/Δ*T*_fn_). The trend changes are more striking on the DMDs.

**Figure 2:**
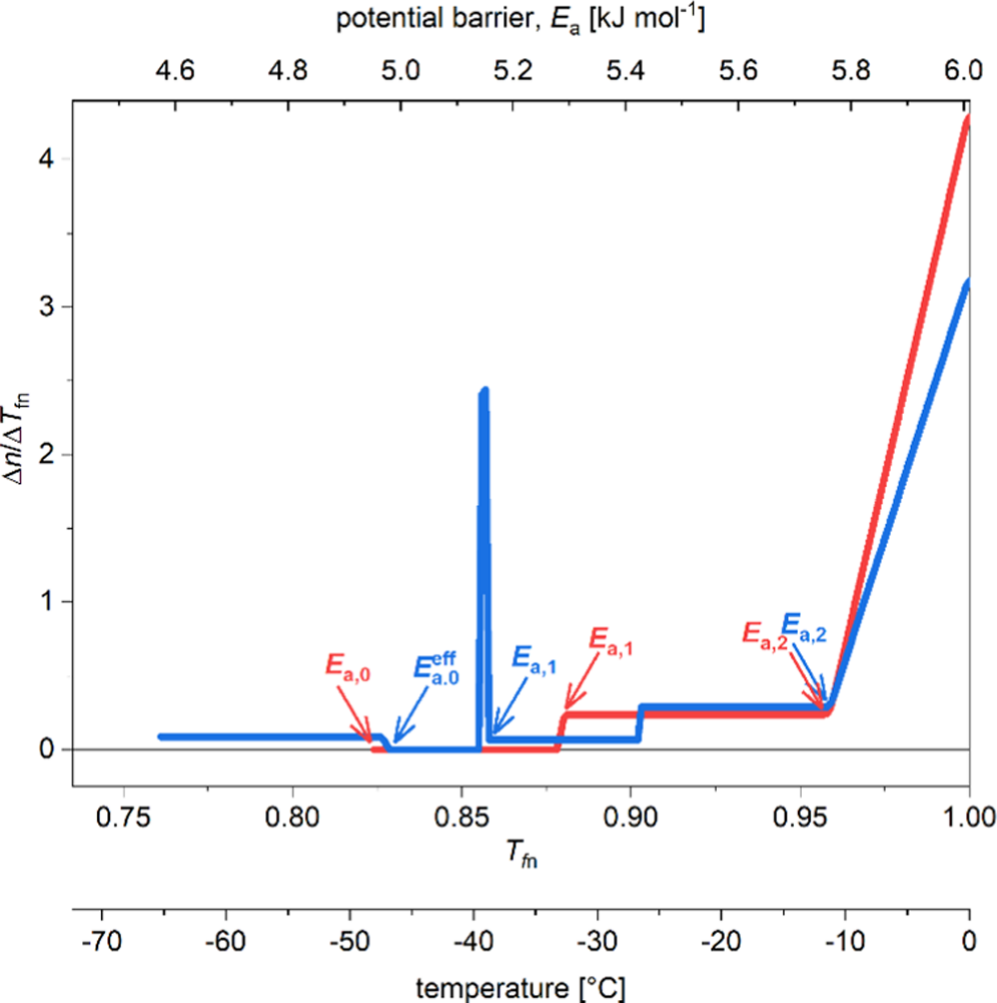
Differential form of the melting diagram of A30P (red) and E46K (blue) α-synuclein. The differentials (Δ*n*/Δ*T*_fn_) were calculated from the fitted curves shown in Figure 1.

There is a short constant *naa* (Figure 1) or zero Δ*n*/Δ*T*_fn_ (Figure 2) section between *E*_a,0_/*E*_a,0_^eff^ and *E*_a,1_ (Table 1) for A30P/E46K. On a microscopic scale, it reflects the presence of *naa* = 0.90(1) mobile hydration with a 4.95(2) kJ mol^–1^ potential barrier regarding the motion of water molecules for A30P. This amount of mobile water corresponds to hydration (g water/g solute) *h* = 0.313(4), which is sufficient for A30P α-S to be active^38,39^ and equals to approximately monolayer hydration^38^ with 126(1) H_2_O/protein. Additional hydration water molecules become mobile at *E*_a,1_, and the amount of mobile hydration increases linearly (constant Δ*n*/Δ*T*_fn_) to reach 277(1) H_2_O/protein at *E*_a,2_ (Table 1). The increase becomes quadratic (linear Δ*n*/Δ*T*_fn_) at *E*_a,2_.

E46K α-S differs from A30P α-S mostly in the low potential barrier region (Figures 1 and 2) and behaves unexpectedly at low potential barrier values. The first mobile hydration water molecules for E46K α-S were detected at a low potential barrier value (*E*_a_ = 4.7(1) kJ mol^–1^), that is, at a low temperature value compared to the other α-S variants (Figure 1 and ref (16)). This potential barrier is as low as in the case of globular proteins (inserted graph in Figure 1).^19,20,36^ The amount of mobile hydration water molecules increases initially, right after the appearance of the first amount of mobile water and before the constant section between *E*_a,0_^eff^ = 4.97 kJ mol^–1^ and *E*_a,1_ = 5.26(2) kJ mol^–1^. The gradual growth of the mobile hydration below *E*_a,0_^eff^ indicates the lack of a step-like change in the potential barriers at *E*_a,0_^eff^ and shows their broad distribution there. The solvent-accessible surface of E46K α-S is very heterogeneous regarding protein–water interactions in this potential barrier section. The plateau (constant *naavsT*_fn_) of E46K has a mobile hydration value of *naa* = 0.335(6) or *h* = 0.117(2), which means 46.9(9) H_2_O/protein, and ends at *E*_a,1_ = 5.26(2) kJ mol^–1^ (Table 1). There is a step, a jump in the magnitude of *naa*, in the MD of E46K at *E*_a,1_ (Figure 1), which corresponds to a spike in the differential form of the MD (DMD, Figure 2). This is followed by a linearly growing section of MD, but with two different regions with different slopes (*B* parameters, Table 1). This corresponds to constant sections with different magnitudes in the DMD (Figure 2). The slope of the MD changes at *E*_a_ = 5.42 kJ mol^–1^. The slope change refers to a change in the interaction between water and E46K α-S at the slope change, *i.e*. at *E*_a_ = 5.42(6) kJ mol^–1^. Different types of water–protein interactions are active below and above the slope change. The hydration at the change is *naa* = 0.8(1) or *h* = 0.27(5) or 2.2(4)·10^–2^ H_2_O/protein. This value is approximately equal to the hydration in the first hydration layer of a protein^38,39^ and the difference between it and *h*(*E*_a,2_) is *h* = 0.33(8) or 2.7(6)·10^–2^ H_2_O/protein. That is, new types of water molecules in their interactions with proteins become mobile at the change additional to the first hydration layer, which becomes built up until the change.

The two mutant α-S variants have nearly parallel MDs at *E*_a,1_ ≤ *E*_a_ ≤ 6.01 kJ mol^–1^ with the A30P α-S having higher mobile hydration (Figure 1). The hydration of A30P α-S is greater by *naa* = 0.35(2) on average than the hydration of E46K α-S between *E*_a,1_^eff^ and *E*_a_ = 6.01 kJ mol^–1^.

The *h*_ho_ and *h*_he_ values, *i.e*., the homogeneously and the heterogeneously bound mobile hydration water amounts for A30P and E46K α-S (Table 2), are markedly lower than in WT and A53T variants (*h*_ho_ = 0.44(8) and *h*_he_ = 2.8(2), on the average).^16^ The A30P and E46K α-S mutants reach their highest hydration level at the melting point of bulk water with an average of *h* = 2.2(3), which corresponds to 9(1)·10^2^ H_2_O/protein. The WT and A53T α-S variants have higher hydration at this point (*h* = 3.31(7) or 1.32(2)·10^3^ H_2_O/protein on the average).^16^

**Table 2:** Dynamic Parameters from the Polynomial Relation Describing the Melting Diagrams^a^

α-S variant	A30P	E46K	A53T	WT
*n*_ho_	0.0156(2)	0.0058(1)	0.22(4)	0.22(4)
*naa*_ho_	0.90(1)	0.335(6)	2.5(5)	2.5(5)
*h*_ho_	0.313(4)	0.117(2)	0.44(8)	0.44(8)
*n*_he_	0.11(2)	0.10(2)	0.18(6)	0.142(9)
*naa*_he_	7(1)	5.5(9)	23(6)	16(1)
*h*_he_	2.3(4)	1.9(3)	4(1)	2.8(2)
*HeR* = (1 – *T*_fn1_)/(1 – *T*_fn0_)	0.68(2)	0.72(3)	0.65(4)	0.70(4)
*HeR_n_* = *n*_he_/(*n*_ho_ + *n*_he_)	0.9(1)	0.9(2)	0.89(3)	0.87(3)
*HeM* = (*B* + 2*C*)/(1 – *T*_fn1_)	8(1)·10^2^	6(1)·10^2^	1.30(6)·10^3^	9.8(5)·10^2^

a For detailed definitions of the parameters, see ref (16). *n*_ho_ = *A* is the mobile water fraction bound homogeneously and *n*_he_ = *n*(*T*_fn_ = 1) – *n*_ho_ is the mobile water fraction bounded heterogeneously. The error in the last digit is given in parenthesis.

Protein molecules can be characterized and categorized by the homogeneity/heterogeneity of the energy distribution of water binding. This ratio is measured and the defining relation is *HeR* = (1 – *T*_fn1_)/(1 – *T*_fn0_), in which (1 – *T*_fn1_) and (1 – *T*_fn0_) give the measured distances from the melting point of ice. The observed heterogeneity ratio for A30P and E46K is *HeR* = 0.69(2) (Table 2), which corresponds to 31(2)% homogeneous potential barrier distribution. This distribution width is equal within experimental error with that of WT and A53T α-S, being 33(4)%.^16^ These homogeneity ratios agree with α-Ss being more compact than it is expected for a random coil state,^40,41^*i.e*., these proteins have a non-negligible extent of secondary structures. The ratio of the amount of heterogeneously bound water to the total number of bound water (heterogeneously plus homogeneously bound) is *HeR_n_* = *n*_he_/(*n*_he_ + *n*_ho_) = 0.9(1) for both A30P and E46K variants, approximately as high as *HeR_n_* = 0.88(1) for WT and A53T variants. The *HeR_n_* values indicate high heterogeneity of the bonds formed by the α-Ss. The measure of heterogeneity, *HeM* = (*B* + 2*C*)/(1 – *T*_fn1_), characterizes the degree of heterogeneity of the protein–water interactions close to 0 °C or *E*_a_ = 6.01 kJ mol^–1^. The A30P and the E46K variants have the same *HeM* values within experimental error, and these values are significantly smaller than those of the WT (9.8(5)·10^2^) and the A53T (1.03(6)·10^3^) variants.^16^ The increase in the hydrations of mutants presented in this work is less intense than in the cases of WT and A53T α-Ss, *HeM*(A53T) > *HeM*(WT) > *HeM*(A30P) ≈ *HeM*(E46K).

### 3.2 *In Silico* Analysis of Secondary Structures

To relate our data from the wide-line NMR results to the disorder and secondary structure content of the two protein variants, we used different *in silico* structure prediction methods.

**Figure 3:**
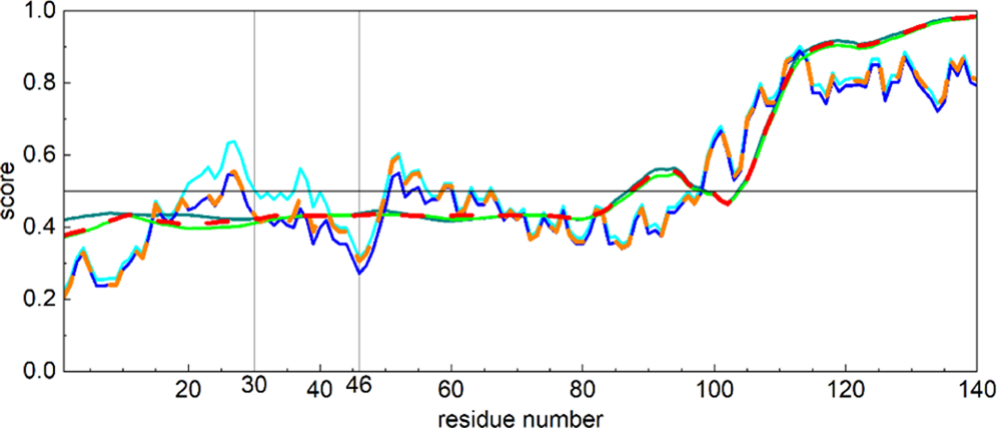
Prediction of protein disorder and disordered binding sites for A30P and E46K α-synuclein by the IUPred2 (A30P cyan, E46K blue solid lines) and ANCHOR2 (A30P dark green, E46K green solid lines) programs. The results for WT α-synuclein are also given for comparison (IUPred2 orange, ANCHOR2 red dashed lines). A score above 0.5 predicts protein disorder or disordered biding site.

Disorder content was predicted with IUPred2,^31,32^ which results in a probability value for each residue of being part of a disordered region, with values above 0.5 indicating disorder tendency. The closer the value is to 1, the higher the probability is of a structurally disordered state at the given position. The average disorder probability scores for the whole length are 0.54(2) for A30P, 0.52(2) for E46K α-S (Figure 3), and 0.53(2) for WT. Between residues 110 to 140, the scores are 0.823(7) and 0.805(8) for A30P and E46K, respectively, while it is 0.819(8) for WT. The first 13 amino acid residues can be considered ordered with a disorder score of 0.29(1) for A30P and 0.27(1) for E46K. This value is 0.27(1) for WT, which equals the latter. The IUPred2A curves of A310P and E46K mutants (Figure 3) coincide with that of WT α-S,^16^ the only exception is A30P between positions 19 and 41, where it shows a higher disorder tendency than the WT and E46K. The ANCHOR2 curve of WT α-S coincides with that of the E46K mutant through the whole protein length and there are only small differences within the first 30 amino acid residues of the A30P mutant (Figure 3). The first 82 residues do not form a disordered binding site (average score of 0.428(2) WT α-S: 4.424(1)) but the last 30 residues at the C terminus show a strong indication for protein binding (score of 0.914(9) WT α-S: 0.926(8)). Residues 100–110 form a transitional region between the two.

Since IUPred provides information on the disorder tendency of a protein but not on the structural propensities of the ordered regions, we also applied *in silico* algorithms to analyze the secondary structure content of the studied proteins.

In the SS predictions, the determinant motifs are coils, helices, and β-sheets according to three- and eight-state prediction methods. Protein secondary structures are traditionally characterized as three general states: helix (H), strand (E), and coil (C). From these general three states, the DSSP^42^ program proposed a finer characterization of the secondary structures by extending the three states into eight states: helix (G), α-helix (H), π-helix (I), β-strand (E), bridge (B), turn (T), bend (S), and others (L). These eight secondary structure states are often mapped into the following three states. H: α-helix, which corresponds to the right- or left-handed cylindrical/helical conformations that include G, H, and I states. E: β-strand, which corresponds to pleated sheet structures that encompass E and B states. C: coil, which covers the remaining S, T, and L states. The state-of-the-art methods are currently reaching almost 88% for a three-class prediction and 76.5% for an eight-class prediction.^43^

**Figure 4:**
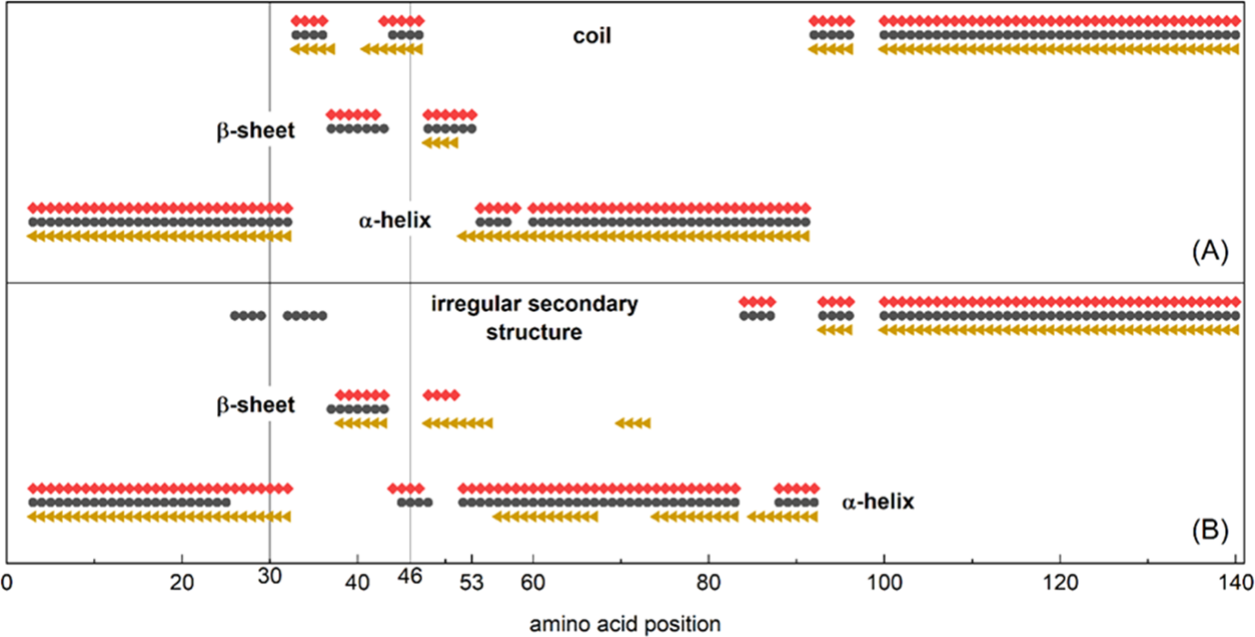
Predicted three- (A) and eight-state (B) secondary structures for A30P (black circles), E46K (red diamonds), and WT (gold triangles) α-synuclein. The average structure of the eight modeling programs for three-state methods (Brewery, Jpred4, Porter 5.0, PSIPRED, PSRSM, RaptorX, SCRATCH, and SPIDER3) and five modeling programs for eight-state methods (Brewery, Porter 5.0, RaptorX, SCRATCH, and SPIDER3) are shown.

For the α-S variants (Figure 4), the three- and eight-state methods indicate the coil and the helix to be the most dominant.

Three-state predictions use the average of the results of eight different SS prediction methods (Brewery, JPred4, Porter 5.0, PSIPRED, PSRSM, RaptorX, SCRATCH, SPIDER3).

The three-state SS prediction of A30P, E46K, and WT α-Ss shows that α-helices and coils are the main SS elements. α-helices form in 47–48% of the protein chain and coils give 39%, while β-sheets are only present in 9%. The β-sheet content is minimal in WT α-S (4 amino acid residues long) but it is three times greater in the A30P (13 residues) and in the E46K (12 residues) mutants.

The eight-state SS prediction consists of the average of five modeling methods (Brewery, Porter 5.0, RaptorX, SCRATCH, SPIDER3). According to this prediction, A30P and E46K mutants contain two longer α-helices (46 and 51% of the whole length, respectively), while the WT version has more pieces of shorter α-helix type sections (43% altogether). The WT α-S in summary shows a longer β-sheet SS (13%) than the two mutants (E46K 7%) and A30P has the shortest (5%) such section.

## 4 Discussion

### 4.1 Structure of the α-Synuclein Mutants

Approximately, one monolayer of mobile water molecules hydrates A30P α-S at the lowest potential barriers (*h* = 0.313(4)). E46K has *h* = 0.117(2) mobile hydration here, which is almost a third of the former and is not enough for the functionality of E46K α-S since a protein needs at least *h* = 0.2 to be functional.^38,44^ The lower initial and then overall hydration can be a result of E46K α-S being more compact than A30P α-S. It appears that every third or fourth hydration site of A30P α-S would be occupied in E46K α-S. The linear hydration section between *E*_a,1_ and *E*_a,2_ for A30P α-S can be described with one slope, while the same section for E46K α-S has two distinct sections with different slopes. The type of the protein–water interaction changes where the slope changes for E46K at *E*_a_ = 5.43(1). *naa* or *h* increases only slightly before the change; it is almost constant here, and after the change, *naa* or *h* increases even more rapidly than A30P α-S. The hydration increases from *E*_a,1_^(eff)^ to *E*_a,2_ are Δ*h*^A30P^ = 0.48(3) and Δ*h*^E46K^ = 0.33(8). This amount of increase suggests a newer layer of mobile hydration to build up.^38^ The rate of expansion of the hydration layers is the greatest at potential barriers greater than *E*_a,2_. The difference in the hydration of A30P and E46K α-Ss in favor of A30P is indicative again of a more compact structure of E46K α-S. The α-S mutants A30P and E46K have markedly lower hydration (*h*(*E*_a_ = 6.01 kJ mol^–1^), Table 1) than the mutant A53T (*h*^A53T^ = 4.0(2)) and the WT (*h*^WT^ = 3.3(2)) α-S at the melting point of bulk water (*E*_a_ = 6.01 kJ mol^–1^). It can be deduced from these data that E46K α-S is the most compact structurally with the smallest solvent-accessible surface and A53T α-S has the most open structure.

A recent cryoelectron microscopy study revealed that the E46K mutant α-S forms structurally distinct, more compact amyloid fibrils than the wild type.^45^ The authors attributed this feature to a misfolding pathway of the mutant, where the salt bridge between E46 and K80 is disrupted by the electrostatic repulsion in the mutant fibril. This altered structural tendency might be reflected already in the monomer form, as indicated by our measurements.

The homogeneously and the heterogeneously bound mobile hydration water amounts for A30P and E46K α-S (*h*_ho_ and *h*_he_, Table 2) also show that the WT and A53T α-Ss are more open structurally than the present mutants.

The heterogeneity ratio, *HeR*, from the dynamic MD parameters shows non-negligible secondary structures in A30P and E46K α-S by the 31(2) % homogeneous potential barrier distribution of the protein–water bonds.

### 4.2 Comparison of the Measured and Predicted Structures

α-S has been mainly considered to contain α-helices with a small number of isolated β-sheets^46−49^ or to be an α + β protein,^50,51^ but the prediction of an all-β structure with some peripheral small α-helices for α-S is also a valid possibility.^52^

Wide-line NMR measurements provide valuable information on the overall solvent accessibility and structural states of the proteins studied but no detailed structural information. Combining our measured data with structure predictions enables us to understand and interpret the structural and physiological consequences of the studied mutations.

IUPred2A^31,32^ is a combined web interface that allows one to identify disordered protein regions using IUPred2 and disordered binding regions using ANCHOR2. The algorithm identifies disordered protein regions and it is found that A30P α-S has longer such regions than E46K α-S. Based on the IUPred results, the disorder tendencies of WT, A30P, and E46K α-Ss are very close to each other, but A30P α-S has a more open structure than E46K α-S. ANCHOR2 predicts regions that undergo a disorder-to-order transition upon binding to another protein. Based on ANCHOR2 results, A30P and E46K, just as WT α-S, have a disordered binding site spanning 30 residues at their C terminus and a transitional but binding region of 10 residues before it.

These results agree with the finding that A30P and E46K α-Ss are also IDPs, as seen by wide-line ^1^H NMR. More precisely, 70(3) % of their solvent-accessible surface is heterogeneous/disordered (Table 2).

Even though the majority of the proteins appears to be in a solvent-accessible, disordered state, they also contain a significant amount of regions with secondary structures.

The three- and eight-state-predicted SSs of the here studied α-S mutants are dominated by α-helices and coils and β-sheets are only minor components of the structures. From this, we can deduce that the secondary structures detected by wide-line NMR are solvent-exposed α-helices in these proteins. The predicted SSs for the three α-S variants (WT, A30P, E46K) are very similar to each other, with the biggest difference being in the extent of β-sheets in the three-state predictions. This method predicts almost no β-sheets in the WT α-S but a markedly increased β-sheet content for the two mutants (Figure 4A). On the contrary, the eight-state predictions forecast the largest β-sheet content for WT α-S, which is in accordance with our earlier secondary structure calculations.^17^

At the sites of the mutations, the predictions show no special features, while the disorder prediction clearly showed an increased disorder tendency of the A30P variant around the mutation site.

A comparison of these predictions with the results of the wide-line NMR reveals that the measured structural states are clearly different from the predicted ones. The mutations induce measurable changes in the secondary structure content of the protein, resulting in alterations of the overall structures in the case of the mutants. This observation also highlights the limits of structure prediction algorithms in detecting structural changes caused by single amino acid changes.

## Accession Codes

WT α-S: P37840; A30P α-S: VAR_007957; E46K α-S: VAR_022703; A53T α-S: VAR_007454; BSA: P02769; β-casein: P02666; lysozyme: P86383.

## Abbreviations

α-S: α-synuclein
BRNN: bidirectional recurrent neural network
DeepCNF: deep convolutional neural fields
DMD: differential melting diagram
DSSP: dictionary of secondary structure of proteins
*HeM*: measure of heterogeneity
*HeR*: heterogeneity ratio
*HeR*_n_: second ratio of heterogeneity
IDP: intrinsically disordered protein
MD: melting diagram
PSI-BLAST: position-specific iterated—BLAST
SS: secondary structure
PD: Parkinson’s disease
WT: wild type
